# Pregabalin dependence, withdrawal, suicidality and psychosis reports: A disproportionality analysis of the Australian adverse events database

**DOI:** 10.1002/bcp.70279

**Published:** 2025-09-26

**Authors:** Amy G. McNeilage, Ali Gholamrezaei, Bridin Murnion, Suzanne Nielsen, Claire E. Ashton‐James

**Affiliations:** ^1^ Sydney Medical School, Faculty of Medicine and Health The University of Sydney Sydney New South Wales Australia; ^2^ Monash Addiction Research Centre, Eastern Health Clinical School Monash University Frankston Victoria Australia; ^3^ School of Clinical Medicine, St Vincent's Healthcare Clinical Campus, Faculty of Medicine and Health University of New South Wales Sydney New South Wales Australia

**Keywords:** adverse drug events, drug dependence, pharmacovigilance, pregabalin, psychiatric disorders

## Abstract

**Aims:**

Globally, Australia has the highest per capita consumption rate of pregabalin, commonly prescribed for neuropathic pain. Emerging evidence suggests pregabalin may be associated with the onset or recurrence of several potentially life‐threatening psychiatric events in some individuals.

**Methods:**

We conducted disproportionality analyses using case reports involving adverse events related to dependence, withdrawal, suicidality and psychosis, submitted to Australia's Therapeutic Goods Administration between 2005 and 2024. Reporting odds ratios (RORs) were calculated to determine whether these adverse events were disproportionately reported for pregabalin compared to all other drugs in the database and to an active comparator group of other neuropathic pain drugs (gabapentin, duloxetine and amitriptyline).

**Results:**

Compared to all other drugs in the database, pregabalin showed strong signals of disproportionate reporting for drug abuse and dependence (ROR = 13.53, 95% confidence interval [CI]: 12.12, 15.10), drug withdrawal (ROR = 6.76, 95% CI: 4.99, 9.15), suicide and self‐injury (ROR = 12.40, 95% CI: 10.71, 14.36), and psychosis and psychotic disorders (ROR = 5.81, 95% CI: 4.99, 6.76). When compared to other neuropathic pain drugs, the signal of disproportionate reporting remained for drug abuse and dependence (ROR = 1.38, 95% CI: 1.17, 1.62) and psychosis and psychotic disorders (ROR = 1.26, 95% CI: 1.002, 1.57), albeit with less pronounced effects.

**Conclusions:**

Adverse events related to drug dependence and psychosis are reported to the Australian pharmacovigilance database at a higher rate for pregabalin compared to other drugs, including other neuropathic pain drugs, signalling a potential concern that warrants further investigation.

**What is already known about this subject**

- Pregabalin prescribing is rising globally, and Australia is the highest per capita consumer.
- Concerns have emerged about its potential association with psychiatric adverse events such as dependence, withdrawal, suicidality and psychosis.
- No studies have analysed pregabalin‐related adverse event reports using Australia's pharmacovigilance database.

**What this study adds**

- Pregabalin is disproportionately associated with reports of dependence, withdrawal, suicidality and psychosis in Australia.
- Signals for dependence and psychosis remain even when compared only to other neuropathic pain drugs.
- The findings highlight the ongoing need for clinical caution, regulatory attention and further research into pregabalin‐related harms.

## 1 INTRODUCTION

Over the past decade, the anticonvulsant pregabalin has been increasingly used in Australia for a diverse range of pain conditions.^1^ An analysis of pharmaceutical sales data from 65 countries found Australia had the highest per capita consumption rate of pregabalin in 2018.^2^ Pregabalin belongs to a class of drugs known as gabapentinoids, which also includes gabapentin. In Australia, the rate of pregabalin consumption is approximately 12 times higher than that of gabapentin,^2^ partly due to differences in government subsidies for these medications.^3^

Pregabalin was first approved by Australia's drug regulation authority—the Therapeutic Goods Administration (TGA)—for the treatment of neuropathic pain and seizures in 2005,^1^ although it is rarely prescribed for seizures.^1^, ^4^ It was then publicly subsidized for refractory neuropathic pain in 2013.^3^ In the years that followed, the rate of prescribing rose considerably. The number of pregabalin scripts dispensed annually in Australia grew from 1.7 million in 2013–2014 to 4 million in 2017–2018,^5^, ^6^ after which it has remained relatively stable.^7^ It now receives more subsidized prescriptions than any other pain drug.^7^

Although the only TGA‐approved indications are neuropathic pain and partial seizures, pregabalin is widely prescribed off‐label, typically for other chronic pain conditions.^1^, ^8^ Primary care records from across Australia show only half of the patients prescribed pregabalin have a recorded diagnosis of neuropathic pain while 1% have a recorded diagnosis of epilepsy.^1^ Given the rapid growth in pregabalin prescribing, it is important to remain vigilant for emerging harms, particularly given its common off‐label use. The side effects most commonly reported in randomized controlled trials (RCTs) of pregabalin include dizziness, somnolence, incoordination and oedema.^9^, ^10^ However, the highly controlled nature of RCTs, including common exclusions of those with mental health conditions, often limits their ability to detect side effects, particularly those that are rare, delayed in onset, or influenced by drug interactions or comorbidities.^11^

Emerging research suggests pregabalin may be associated with the onset or recurrence of a number of potentially life‐threatening psychiatric events. Over the past decade, Australian studies have reported increases in pregabalin‐related ambulance attendances, poisonings and deaths.^12^, ^13^ Moreover, research from around the world indicates a clear risk of misuse and dependence with pregabalin.^14^, ^15^ Concerningly, evidence emerging from qualitative research^15^ and case studies^16^, ^17^, ^18^, ^19^ suggests that suicide‐related behaviours, self‐harm and psychosis may also be associated with pregabalin use and discontinuation in some patients.

Pharmacovigilance databases that collect reports of suspected adverse events (AEs) from healthcare providers, consumers and pharmaceutical companies can provide valuable insights into drug safety concerns.^20^ A common method for analysing AE reports is a disproportionality analysis, which tests whether the reporting rate of a drug–event combination of interest is disproportionately higher than the rate in a comparator drug or set of drugs.^20^, ^21^ Disproportionality analyses are predominantly exploratory, hypothesis‐generating studies to detect signals of disproportionate reporting (SDRs) that warrant further exploration in pharmacoepidemiology and clinical trials.^22^ They can also be useful for testing working hypotheses before designing more rigorous and resource‐intensive studies.^23^

To our knowledge, no studies have explored pregabalin AE reports using data from the Australian pharmacovigilance database. Given its high use rates and robust regulatory framework, the Australian context is well suited to studying emerging pregabalin‐related harms. The aim of this study was to characterize and analyse AEs attributed to pregabalin using spontaneous and voluntary reports submitted to the Australian pharmacovigilance database. There were 3 specific objectives: first, to describe the AEs including deaths attributed to pregabalin over the past 2 decades; second, to conduct a disproportionality analysis to determine whether there were SDRs for any of the AEs of interest (i.e., drug dependence and withdrawal, suicidal ideation and self‐injury, and psychosis) for pregabalin when compared to all drugs in the database; and third, to conduct active‐comparator restricted disproportionality analyses of the AEs of interest to determine whether there were SDRs for pregabalin when compared to clinically comparable drugs (i.e., gabapentin, duloxetine and amitriptyline).

## 2 METHODS

### 2.1 Study design

This was a retrospective observational study using AE case reports submitted to the Australian pharmacovigilance database. The findings herein are reported according to The REporting of A Disproportionality Analysis for DrUg Safety Signal Detection Using Individual Case Safety Reports in PharmacoVigilance (READUSPV) statement (see Supporting Information for completed READUSPV checklist).^20^ In line with pharmacovigilance research recommendations,^22^ the design, conduct and interpretation of this disproportionality analysis involved a multidisciplinary team of clinicians, researchers and statisticians.

### 2.2 Data source

Data were sourced from the TGA's Database of Adverse Event Notifications (DAEN; https://daen.tga.gov.au/medicines-search/). The DAEN is publicly accessible and includes spontaneous and voluntary case reports of AEs (sometimes referred to as suspected adverse drug reactions or suspected side effects) about medicines used in Australia. The TGA defines an AE as any unfavourable and unintended sign, symptom or disease believed to be associated with the use of a medicine. In Australia, healthcare providers and consumers are encouraged to reports AEs, while pharmaceutical companies are required by law to report serious AEs.

Case reports are submitted to the TGA via a standardized electronic form.^24^ Missing details may be clarified with the reporter before the case is coded and entered into the internal Adverse Event Management System. All AEs are coded using Medical Dictionary for Regulatory Activities (MedDRA) preferred terms.^25^ The MedDRA is a hierarchical taxonomy with >26 000 terms used internationally. Medicines are recorded by trade names and active ingredients. Two weeks after entry into the Adverse Event Management System, deidentified reports are published in the public DAEN with the following details: case number, report date, age, sex, suspected and not suspected medicines reported as being taken, and MedDRA terms. Unlike some other pharmacovigilance databases, the public DAEN does not provide information on reporter type or therapeutic indication. The DAEN contains >630 000 case reports dating back to 1971.

### 2.3 Comparator drugs

Given the complex nature of selecting appropriate comparators in disproportionality analyses,^26^ we opted for a 2‐stage approach.^22^ First, we used the full dataset (i.e., all drugs in the database) as the comparator group to establish a benchmark for the broader reporting landscape and to identify potential SDRs. While full dataset comparisons are generally more robust against reporting biases,^21^ they do not account for confounding by indication. This is particularly relevant given that chronic neuropathic pain—the primary indication for pregabalin—is associated with an increased rate of psychiatric comorbidities such as depression, anxiety and suicidal ideation.^27^, ^28^ To address this limitation, the second stage of our analysis used an active comparator approach, restricting comparisons to drugs with similar indications or therapeutic uses.^26^ Active comparator analyses can help reduce false positive signals and mitigate channelling bias.^29^ The use of both full‐dataset and active comparator analyses has been recommended to strengthen signal assessment and contextualize findings.^22^

While we considered the use of negative controls to assess the specificity of our approach, identifying a valid negative control in the context of psychiatric outcomes and chronic pain is challenging. Many drugs that could serve as candidate comparators (e.g., paracetamol, nonsteroidal anti‐inflammatory drugs, opioids) are themselves associated with overdose, self‐harm, or other psychiatric adverse events, making them unsuitable as true negatives. Given these constraints, we elected not to include a negative control and instead relied on the combination of full‐dataset and active comparator analyses to provide a balanced assessment and reduce bias.

Both Australian and international guidelines recommend gabapentin, duloxetine and amitriptyline, alongside pregabalin, as first‐line medicines for the treatment of neuropathic pain.^30^, ^31^ In addition, these drugs have been used as comparators in other studies of pregabalin AE reports internationally,^32^, ^33^, ^34^, ^35^ allowing for comparisons across studies. Case reports associated with the active comparator drugs—gabapentin, duloxetine and amitriptyline—were grouped together as a set labelled *other neuropathic pain drugs*. We chose to group the therapeutic alternatives instead of using a single comparator drug to reduce the risk of overfitting or detecting spurious effects.

The comparator drugs were chosen in discussion with a pharmacist (S.N.) and physician specializing in pain medicine, addiction and clinical pharmacology and toxicology (B.M.). Gabapentin is in the same class of drugs to pregabalin (i.e., gabapentinoids) and has a similar mechanism of action. Duloxetine (a serotonin–norepinephrine reuptake inhibitor) and amitriptyline (a tricyclic antidepressant) have distinct mechanisms. Gabapentin is TGA‐registered for neuropathic pain, duloxetine is TGA‐registered for diabetic peripheral neuropathic pain, and amitriptyline is used off‐label for neuropathic pain in accordance with guidelines. Like pregabalin, gabapentin is also indicated for partial seizures, while duloxetine and amitriptyline are indicated for major depressive disorder. In Australia during 2023–24, pregabalin was dispensed in 4 043 890 prescriptions, compared to 2 862 712 for amitriptyline and 2 261 367 for duloxetine.^7^ Prescription volumes for gabapentin were not reported in the Pharmaceutical Benefits Scheme (PBS) summary data as it was not among the top 50 dispensed medicines.^7^

### 2.4 Data extraction

All AE case reports for pregabalin, gabapentin, duloxetine and amitriptyline submitted between 1 April 2005 and 31 December 2024 were downloaded from the DAEN in an Excel spreadsheet (a list of all trade names included for each drug is available in the Supporting Information). The 20‐year study period corresponds with the timeframe in which pregabalin has been approved for neuropathic pain treatment in Australia, capturing all available AE reports for pregabalin in the database. As individual case reports in the public DAEN do not include information on patient outcomes, data on deaths were extracted from the DAEN summary tables, which list the number of reports with an outcome of death for each suspected medicine. These data reflect the number of cases where pregabalin (or comparator drugs) was listed among suspected medicines in a report with a fatal outcome; they do not establish causality. Data used for the full dataset comparisons were also extracted from the DAEN in summary form.

### 2.5 Data cleaning and transformation

The TGA seeks to identify and consolidate duplicate cases through reporting guidelines and internal processes,^36^ though some risk remains. Case reports listing multiple comparator drugs were counted once in comparator set. Age and sex were the only variables with missing data, and these cases remained in the analysis. MedDRA terms relating to our AEs of interest were re‐coded according to Standardized MedDRA Queries (SMQs). These SMQs are validated sets of AE terms developed by MedDRA through extensive review, testing, analysis and expert discussion.^25^ We used the broad scope terms for the following SMQs that mapped on to our AEs of interest: (i) drug abuse and dependence; (ii) drug withdrawal; (iii) suicide and self‐injury; and (iv) psychosis and psychotic disorders. Broad terms include both narrow terms and other related terms that might identify potential cases or early signs, but with less certainty.^25^ Lists of all the terms included for each SMQ are provided in the Supporting Information. The AEs of interest were selected a priori predominantly on the basis of our research team's recent systematic review of qualitative research,^15^ as well as literature outlined in the introduction. The following variables were created for each case using extracted data: total number of AEs reported; other medicines reported as being taken (both suspected and not suspected); and the presence of a suspected drug interaction. Cases were classified as *single suspected* if only 1 active ingredient was involved, regardless of how many trade names were reported.

### 2.6 Data analysis

Descriptive statistics were calculated for all AE reports and deaths where pregabalin or an active comparator drug was listed as a suspected medicine. For continuous variables, means and standard deviations were computed, with medians and interquartile ranges preferentially reported for non‐normally distributed data. For categorical variables, frequencies and proportions were reported.

Disproportionality analyses were performed to determine whether pregabalin was disproportionately associated with reporting of the AEs of interest. When at least 3 reports were recorded, disproportionality was assessed using the reporting odds ratio (ROR) with 95% confidence intervals (CIs). Proportional reporting ratios (PRRs) were also calculated as a robustness check; however, as results were highly similar and did not alter the significance of findings, only ROR values are presented in the main text, with PRR provided in the Supporting Information. Signals of disproportionate reporting were identified when the lower bound of the 95% CI exceeded 1. For ROR, values <1 suggested a lower likelihood of reporting for the drug‐AE pair compared to the comparator drug or set. The ROR and PRR were calculated according to standard formulas, with case counts defined in the Supporting Information.

Sensitivity analyses were performed on the active comparator analyses by adjusting the comparator to determine whether specific drugs contributed to observed SDRs. In these analyses, the same formulas for ROR and PRR were applied. Additional sensitivity analyses explored potential confounders, including: (i) subgroup analyses by age (18–34, 35–44, 45–54, 55–64, >65 years), sex (male, female) and reporting period (2005–2009, 2010–2014, 2015–2019, 2020–2024); (ii) restricting the SMQs to narrow terms only; and (iii) limiting the dataset to cases with a single suspected medicine. All analyses were conducted using JASP,^37^ and a *P*‐value <.05 was considered statistically significant.

### 2.7 Nomenclature of targets and ligands

Key protein targets and ligands in this article are hyperlinked to corresponding entries in http://www.guidetopharmacology.org and are permanently archived in the Concise Guide to PHARMACOLOGY 2023/24.^38^, ^39^

## 3 RESULTS

The DAEN contained 460 528 AE case reports submitted between 1 April 2005 and 31 December 2024. Pregabalin, gabapentin, duloxetine and amitriptyline were suspected in 2016 cases, 426 cases, 1008 cases and 573 cases, respectively.

### 3.1 Features of pregabalin AE case reports

**FIGURE 1:**
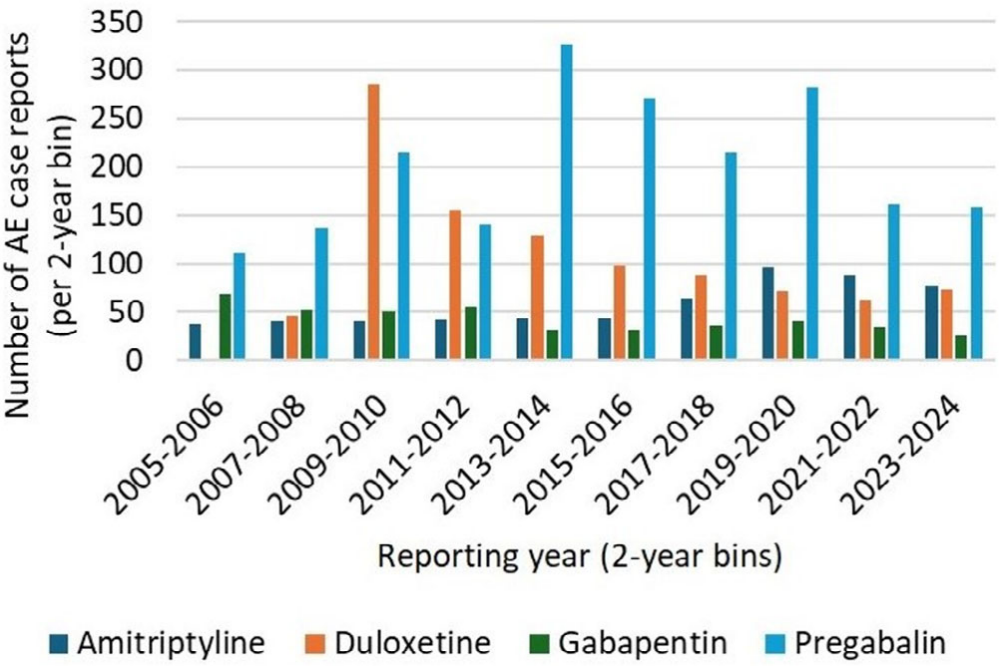
Number of adverse event case reports by drug (pregabalin, gabapentin, duloxetine, amitriptyline) in 2‐year intervals, 2005–2024. Bars show the number of case reports in each 2‐year period. The y‐axis represents counts; this figure does not present a reporting rate because exposure denominators (e.g., prescription volumes) were unavailable.

The mean age of individuals involved in pregabalin‐related case reports was 55.5 years (standard deviation = 18.5), and the majority were female. The mean age and sex distribution for the comparator drugs are reported in Table 1. Of the 2016 pregabalin‐related cases, pregabalin was the sole suspected drug in 72% (*n* = 1455), 1 of multiple suspected drugs in 27% (*n* = 542) and part of a suspected drug interaction in 1% (*n* = 19). Pregabalin AE reports peaked in 2013–2014 and again in 2019–2020, followed by a decline (Figure 1). Pregabalin case reports involved 6131 AEs in total (M = 3.0 per case, standard deviation = 4.0), comprising 1045 unique terms. The most common AEs were toxicity to various agents (included in 10% of all pregabalin case reports), suicidal ideation (7%) and overdose (7%). Toxicity, dizziness and nausea were among the top 10 AEs across pregabalin and all comparator drugs (Table 2).

**TABLE 1:** Characteristics of individuals involved in adverse event case reports for pregabalin, gabapentin, duloxetine and amitriptyline.

Variable	Pregabalin (*N =* 2016)	Gabapentin (*N =* 426)	Duloxetine (*N =* 1008)	Amitriptyline (*N =* 573)
Age, years				
M (SD)	55.5 (18.5)	56.3 (19.9)	49.2 (17.2)	51.5 (19.2)
Missing (%)	736 (36.5)	144 (33.8)	278 (27.6)	145 (25.3)
Sex				
Female (%)	1133 (56.2)	264 (62.0)	644 (63.9)	334 (58.3)
Male (%)	801 (39.7)	141 (33.1)	319 (31.7)	215 (37.5)
Missing (%)	82 (4.1)	21 (4.9)	45 (4.5)	24 (4.2)

Abbreviation: SD, standard deviation.

**TABLE 2:** Top 10 most frequently reported adverse events for pregabalin, gabapentin, duloxetine and amitriptyline.

No.	Pregabalin	*N*	Gabapentin	*N*	Duloxetine	*N*	Amitriptyline	*N*
1	Toxicity to various agents	202	Drug ineffective	38	Nausea	121	Toxicity to various agents	127
2	Suicidal ideation	144	Dizziness	33	Dizziness	85	Serotonin syndrome	38
3	Overdose	140	Nausea	32	Headache	68	Confusional state	35
4	Dizziness	136	Somnolence	32	Toxicity to various agents	67	Overdose	32
5	Somnolence	133	Fatigue	24	Suicidal ideation	66	Dry mouth	29
6	Confusional state	86	Headache	19	Insomnia	60	Somnolence	27
7	Vision blurred	83	Suicidal ideation	18	Withdrawal syndrome	56	Nausea	25
8	Weight increased	82	Toxicity to various agents	16	Vomiting	55	Dizziness	25
9	Nausea	80	Memory impairment	16	Serotonin syndrome	50	Drug ineffective	24
10	Depression	78	Depression	16	Diarrhoea	49	Drug interaction	22

Individuals reporting pregabalin AEs were taking a median of 2 medicines (interquartile range: 1–5). The most frequently reported comedications were paracetamol (in 13% of cases), oxycodone (11%) and diazepam (8%). In pregabalin cases with multiple suspected medicines, the most common other *suspected* medicines were diazepam (18%), oxycodone (17%) and amitriptyline (14%). Among the 19 interaction cases, oxazepam (32%), diazepam (26%) and amitriptyline (21%) were most frequently implicated.

### 3.2 Suspected pregabalin‐related deaths

Pregabalin was suspected in 345 reported deaths. The most common AEs in these cases were toxicity to various agents (54%), overdose (34%), pneumonia (10%), accidental overdose (10%) and suicide (7%). By comparison, there were 15, 92 and 147 suspected deaths involving gabapentin, duloxetine and amitriptyline, respectively.

### 3.3 Full‐dataset disproportionality analyses

Table 3 presents the proportion of case reports involving each AE of interest for pregabalin and other drugs. When compared to all other drugs in the database, pregabalin showed strong SDRs for drug abuse and dependence (ROR = 13.53, 95% CI: 12.12, 15.10), drug withdrawal (ROR = 6.76, 95% CI: 4.99, 9.15), suicide and self‐injury (ROR = 12.40, 95% CI: 10.71, 14.36) and psychosis and psychotic disorders (ROR = 5.81, 95% CI: 4.99, 6.76).

**TABLE 3:** Number and proportion of case reports involving adverse event (AEs) of interest.

AE of interest	Pregabalin (*N =* 2016)		Gabapentin (*N =* 426)		Duloxetine (*N =* 1008)		Amitriptyline (*N =* 573)		All drugs (*N =* 460 528)	
	*n*	%	*n*	%	*n*	%	*n*	%	*n*	%
Drug abuse and dependence	418	20.7	35	8.2*	126	12.5*	167	29.1*	9116	2.0*
Drug withdrawal	44	2.2	7	1.6	76	7.5*	7	1.2	1553	0.3*
Suicide and self‐injury	208	10.3	26	6.1*	124	12.3	45	7.9	4423	1.0*
Psychosis and psychotic disorders	187	9.3	38	8.9	61	6.1*	53	9.3	8121	1.8*

*Note*: * The proportion is significantly different from pregabalin, *P* < .05.

### 3.4 Active comparator disproportionality analyses

While the grouped comparator set formed the basis of our primary analyses (see Table 4), results stratified by individual comparator drugs are presented in Table 5 as sensitivity analyses to explore whether findings were consistent across drugs.

**TABLE 4:** Results of active comparator disproportionality analyses for adverse event (AEs) of interest.

AE of interest	Pregabalin	Other neuropathic pain drugs
	ROR (95% CI)	ROR (95% CI)
Drug abuse and dependence	1.38 (1.17, 1.62)*	REF
Drug withdrawal	0.46 (0.32, 0.67)*	REF
Suicide and self‐injury	1.09 (0.88, 1.34)	REF
Psychosis and psychotic disorders	1.26 (1.002, 1.57)*	REF

*Note*: * indicates *P* < .05. REF = reference set. Other neuropathic pain drugs set includes gabapentin, duloxetine and amitriptyline combined.

Abbreviations: CI, confidence interval; ROR, reporting odds ratio.

**TABLE 5:** Results of sensitivity analyses using different comparators.

AE of interest	Comparator drug	Pregabalin
		ROR (95% CI)
Drug abuse and dependence	Gabapentin	2.92 (2.03, 4.20)*
	Duloxetine	1.83 (1.48, 2.27)*
	Amitriptyline	0.64 (0.52, 0.78)*
Drug withdrawal	Gabapentin	1.34 (0.60, 3.00)
	Duloxetine	0.27 (0.19, 0.40)*
	Amitriptyline	1.80 (0.81, 4.03)
Suicide and self‐injury	Gabapentin	1.77 (1.16, 2.70)*
	Duloxetine	0.82 (0.65, 1.04)
	Amitriptyline	1.35 (0.96, 1.89)
Psychosis and psychotic disorders	Gabapentin	1.04 (0.72, 1.51)
	Duloxetine	1.59 (1.78, 2.14)*
	Amitriptyline	1.00 (0.73, 1.38)

*Note*: * indicates *P* < .05.

Abbrevations: CI, confidence interval; ROR, reporting odds ratio.

#### 3.4.1 Drug abuse and dependence AEs

The disproportionality analysis detected increased reporting of drug abuse and dependence AEs for pregabalin compared to other neuropathic pain drugs (Table 4). The finding held for both cases with a single suspected medicine (ROR = 2.37, 95% CI: 1.76, 3.19) and cases with multiple suspected medicines (ROR = 1.67, 95% CI: 1.33, 2.09). However, the signal was not significant when using narrow SMQ terms (ROR = 1.37, 95% CI: 0.99, 1.90).

**FIGURE 2:**
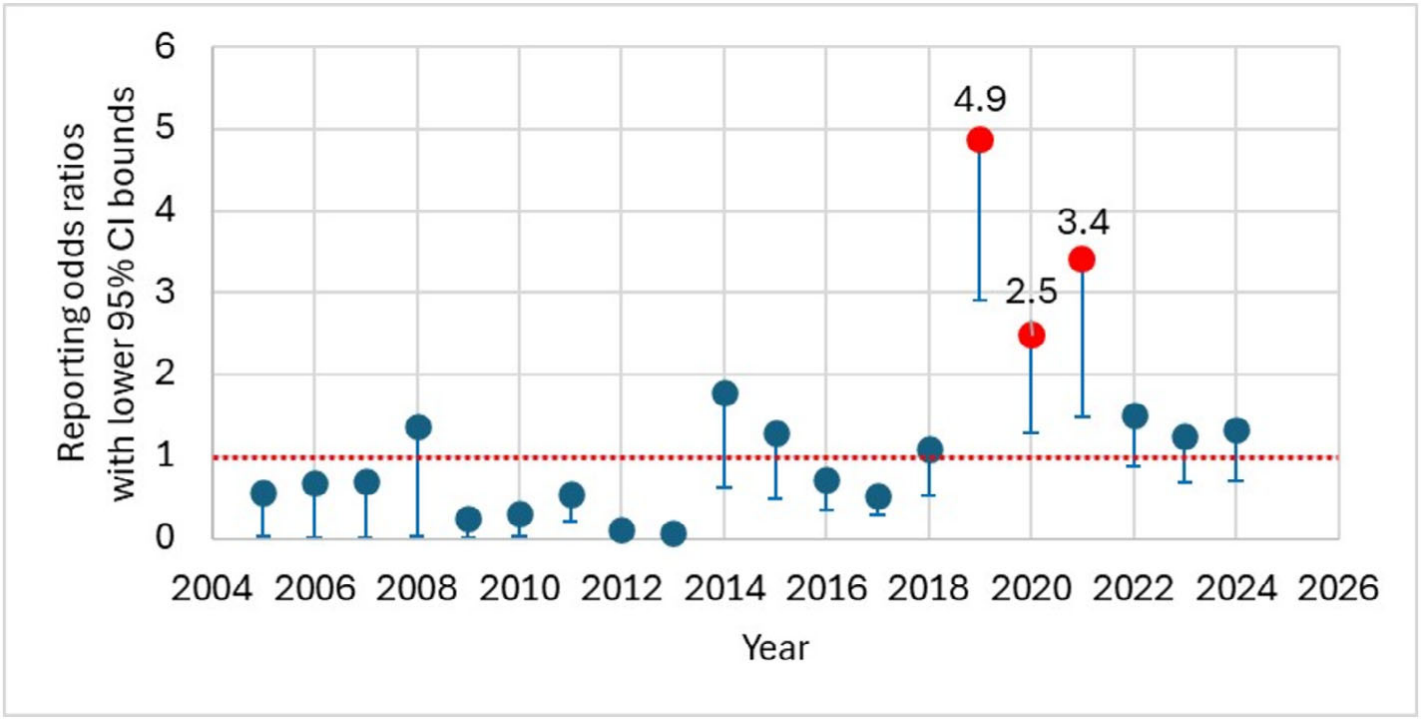
Annual reporting odds ratios for drug abuse and dependence adverse events associated with pregabalin compared to other neuropathic pain drugs. Note: points represent the central estimate, and error bars extend to the lower bound of the 95% confidence interval. The horizontal reference line at y = 1 indicates the null value, with values above 1 suggestive of a potential disproportionality signal.

Adjusting the comparator (Table 5) produced stronger signals when gabapentin or duloxetine were used, but decreased reporting compared to amitriptyline. Increased reporting was seen only in the 35–44 age group (ROR = 2.16, 95% CI: 1.38, 3.38), while decreased reporting was observed in the over 65 group (ROR = 0.37, 95% CI: 0.21, 0.67). Additionally, the signal was significant in males (ROR = 1.87, 95% CI: 1.46, 2.40), but not females (ROR = 1.09, 95% CI: 0.87, 1.37). Time‐specific analyses showed stronger signals in 2015–2019 (ROR = 1.47, 95% CI: 1.11, 1.94) and 2020–2024 (ROR = 1.68, 95% CI: 1.27, 2.20), with RORs peaking in 2019, 2020 and 2021 (Figure 2). In contrast, decreased reporting was observed in 2010–2014 (ROR = 0.45, 95% CI: 0.27, 0.74), and there were not enough cases in 2005–2009 to support analysis.

#### 3.4.2 Drug withdrawal AEs

The disproportionality analysis showed decreased reporting of drug withdrawal AEs for pregabalin compared to other neuropathic pain drugs (Table 4), though the difference was not significant when using narrow SMQ terms (ROR = 0.60, 95% CI: 0.32, 1.15). Comparator adjustment showed reduced reporting compared to duloxetine only; no differences were found for gabapentin or amitriptyline (Table 5). The results of all sensitivity analyses for AEs of interest are available in the Supporting Information.

#### 3.4.3 Suicide and self‐injury AEs

The disproportionality analysis did not detect a difference in the rate of reporting of suicide and self‐injury AEs for pregabalin compared to other neuropathic pain drugs (Table 4). There was increased reporting when gabapentin was used as the comparator, but no difference when compared to either duloxetine or amitriptyline (Table 5). The suicide and self‐injury SMQ includes narrow terms only.

#### 3.4.4 Psychosis and psychotic disorder AEs

The disproportionality analysis detected increased reporting of psychosis and psychotic disorder AEs for pregabalin compared to other neuropathic pain drugs (Table 4), although the SDR was only narrowly above the threshold for significance and should therefore be interpreted with caution. The SDR was stronger when using narrow SMQ terms (ROR = 1.40, 95% CI: 1.05, 1.88). While the SDR was significant in cases with a single suspected medicine (ROR = 1.44, 95% CI: 1.10, 1.90), it was not in cases with multiple suspected medicines (ROR = 0.74, 95% CI: 0.47, 1.16). Increased reporting was observed with duloxetine as comparator, but not with gabapentin or amitriptyline (Table 5).

## 4 DISCUSSION

To our knowledge, this is the first study to systematically describe and analyse AE case reports for pregabalin in the Australian pharmacovigilance database. We identified strong SDRs for dependence, withdrawal, suicidality and psychosis when compared to all other drugs. Signals for dependence and psychosis remained when compared to other neuropathic pain drugs, although effect sizes were smaller. These findings are best understood in relation to existing evidence and clinical concerns.

Most of the commonly reported AEs in this study—such as dizziness, somnolence, confusional state, blurred vision, weight gain and nausea—are well known to be associated with pregabalin.^10^ However, the top 3 AEs in our data—toxicity to various agents (i.e., harmful effects from exposure to multiple substances), suicidal ideation and overdose—are not typically observed in RCTs,^10^ which often evaluate pregabalin as a monotherapy and exclude patients with psychiatric comorbidities or substance‐use histories.^10^ In contrast, our study draws on real‐world data and offers insights into AEs that may not be captured in RCT settings. That said, serious AEs such as suicidal ideation may be more likely to be reported to pharmacovigilance databases than less serious events, potentially inflating their apparent relative risk.

Toxicity to various agents was the AE most commonly implicated in pregabalin‐related deaths. This is consistent with prior research showing the vast majority of pregabalin‐related deaths in Australia involve other substances, particularly opioids.^12^, ^40^ Fatal overdoses involving pregabalin alone are rare.^40^ Despite this, pregabalin is often coprescribed with opioids in Australia,^1^, ^4^ significantly increasing the risk of overdose.^41^ This raises important questions about whether patients and prescribers are fully aware of these risks or have access to safer alternatives. Regardless, the additive dangers of pregabalin–opioid coprescribing demand careful clinical monitoring, especially at higher doses.

Our full dataset analyses detected strong SDRs for all AEs of interest. Similar patterns have been observed in pharmacovigilance data from the USA.^42^, ^43^ It is possible that increased reporting of these AEs may reflect the underlying vulnerability of the patient population (i.e., individuals with neuropathic pain) rather than the safety profile of pregabalin itself. To account for this, we also compared pregabalin to other drugs commonly used for neuropathic pain (gabapentin, duloxetine and amitriptyline). In these analyses, pregabalin showed higher reporting rates for dependence and psychosis AEs, lower rates for withdrawal AEs and no significant difference for suicidality.

Sensitivity analyses indicated that the SDR for drug abuse and dependence was largely driven by the inclusion of gabapentin in the comparator set. Increased reporting of drug abuse and dependence AEs for pregabalin compared to gabapentin has also been observed in both US and European pharmacovigilance data.^34^, ^35^ Despite their similar mechanisms of action, evidence points to dependence risks being more pronounced for pregabalin than gabapentin.^34^, ^35^, ^44^, ^45^, ^46^ This difference has been attributed to pregabalin's faster onset of action, increased bioavailability at high doses and nonsaturable absorption.^47^, ^48^ Although direct comparisons of the abuse liability of the 2 drugs are limited, studies have shown that people who use drugs recreationally report significantly higher subjective ratings of *drug liking*, *good drug effects* and feeling *high* after taking a therapeutic dose of pregabalin (200 mg) compared to placebo.^49^ At supratherapeutic doses (450 mg), these ratings are comparable to those for 30 mg of diazepam.^49^, ^50^ Our findings add to a growing body of evidence indicating that pregabalin carries risks of misuse and dependence that warrant careful consideration and monitoring by prescribers.^14^, ^15^ However, this study was not designed to assess the relative safety of pregabalin compared to other neuropathic pain drugs, and more rigorous studies are needed before drawing conclusions that can guide clinical decision‐making.

Although the observed SDR for psychosis was of borderline significance, it raises important concerns given the serious nature of these conditions and their implications for patient safety and wellbeing. This finding should also be interpreted in light of the frequent coreporting of centrally acting drugs, particularly opioids (e.g., oxycodone) and benzodiazepines (e.g., diazepam), which may have contributed to the observed reporting patterns. In contrast, our other active comparator analyses detected decreased reporting of drug withdrawal AEs, and no difference in reporting for suicide and self‐injury AEs. However, the absence of a signal should not be interpreted as evidence of safety. Shared AE profiles among neuropathic pain drugs may mask true risks.^22^ For example, duloxetine and amitriptyline are known to be associated with withdrawal and mood‐related AEs.^51^, ^52^, ^53^, ^54^ In addition, these drugs are used to treat major depressive disorder, meaning that the populations prescribed them may be inherently more vulnerable to psychiatric events than those prescribed pregabalin. Prescribers may also preferentially prescribe specific neuropathic pain drugs (i.e., antidepressants) to patients with psychiatric histories, further complicating comparisons. While this study cannot definitively confirm or disconfirm any associations, it does underscore the need to better understand the nature of psychiatric AEs among individuals prescribed pregabalin, particularly in real‐world settings that reflect diverse patient populations.

### 4.1 Limitations

The reporting of AEs to the DAEN is voluntary, spontaneous and not independently verified. Reports often lack clinical context, such as whether the AE occurred during prescribed use, misuse, or discontinuation. This limitation is especially relevant for AEs related to dependence or withdrawal, where the pattern of use might affect both the occurrence and severity of the event, as well as the likelihood of it being reported. In addition, the public DAEN does not provide details on reporter type (e.g., clinician, consumer, company) or on the therapeutic indication for which the medicine was used. The absence of this information limits our ability to assess whether reporting patterns differed by reporter group or to distinguish on‐label from off‐label use.

While a signal detected through disproportionality analysis indicates a significant difference in the rate of reporting, it does not constitute evidence of causation. Other factors such as reporting bias and confounding can influence the presence and strength of a signal. Healthcare providers are known to under‐report AEs,^55^ potentially due to time constraints, limited awareness of reporting processes, or perceptions about the clinical relevance of the event. Conversely, when AEs are self‐reported by patients, a lack of clinical knowledge may affect the accuracy or validity of the report. External factors—such as time on market, media attention or public discourse around safety concerns—can also influence reporting behaviour.^21^ These influences partly motivated our time‐based sensitivity analysis which showed that SDRs for drug abuse and dependence AEs were strongest in 2019, 2020 and 2021, a period predating a TGA safety warning concerning the potential risks of abuse and dependence.^56^

With regards to confounding, reported AEs and deaths may be attributable to the underlying condition, comorbidities or other medications rather than the drug of interest. We sought to mitigate some of this confounding by using an active comparator. However, it is not possible to fully account for baseline differences in the populations prescribed different drugs. Despite these limitations, previous research has shown that relative risks obtained from disproportionality analyses often correlate with those from meta‐analyses.^57^ It is also important to note that raw AE reporting counts should not be compared directly across drugs without considering differences in prescribing rates. For example, pregabalin is more commonly prescribed in Australia than the comparator drugs. Measures such as RORs and PRRs, which reflect proportional rather than absolute reporting, offer more meaningful insights into relative risk. Although we considered calculating crude reporting rates using PBS dispensing data, these were not included because PBS data do not capture all prescriptions (e.g., private or hospital supply), under‐capture low‐cost drugs prior to 2012, and do not provide denominators for gabapentin.

Finally, only frequentist disproportionality methods (specifically ROR and PRR) were applied in this study. While the main drug–event pairs analysed were supported by hundreds of reports, sensitivity and subgroup analyses involved smaller case counts, where frequentist approaches may yield less stable estimates. Bayesian shrinkage methods may provide more robust signal detection in such contexts and could be considered in future work.^58^, ^59^

## 5 CONCLUSION

Drug dependence and psychosis AEs are reported to the Australian pharmacovigilance database at an increased rate for pregabalin compared to other drugs, including other neuropathic pain drugs, signalling a potential concern warranting further investigation. The findings of this study add to the growing body of evidence indicating that there are psychiatric risks associated with pregabalin that should be carefully considered and monitored by prescribers. However, due to potential reporting biases and confounding, the safety of pregabalin relative to other neuropathic pain drugs cannot be established from this study alone. Further robust, prospective research is needed to guide clinical decision‐making.

## AUTHOR CONTRIBUTIONS

Conceptualisation: Amy G. McNeilage. Project administration: Amy G. McNeilage, Ali Gholamrezaei. Methodology: all authors. Data curation: Ali Gholamrezaei. Formal analysis and investigation: Amy G. McNeilage, Ali Gholamrezaei. Writing—original draft: Amy G. McNeilage. Writing—review and editing: all authors. Visualization: Amy G. McNeilage, Ali Gholamrezaei. Supervision: Birdin Murnion, Suzanne Nielsen, Claire E. Ashton‐James. Funding acquisition: Amy G. McNeilage, Suzanne Nielsen, Claire E. Ashton‐James. All authors read and approved the final version.

## CONFLICT OF INTEREST STATEMENT

The authors have no relevant financial or nonfinancial interests to disclose.

## ETHICS APPROVAL

As the research was considered low risk and used only publicly available de‐identified data, the University of Sydney Human Research Ethics Office advised it was exempt from ethics review.

## Supporting information


**DATA S1** Supporting information

**DATA S2** Supporting information

## Data Availability

The data that support the findings of this study are available from the corresponding author upon reasonable request.
