# Supplementary material for: Pregabalin dependence, withdrawal, suicidality and psychosis reports: A disproportionality analysis of the Australian adverse events database
Source: Br J Clin Pharmacol. 2025 Sep 26;92(2):545–55. doi: 10.1002/bcp.70279 (PMC12850631; doi:10.1002/bcp.70279)
Supplement: Supplementary file 1 — DATA S1 Supporting information [file BCP-92-545-s002.docx]

**Supporting Information**

**Article title:** Pregabalin dependence, withdrawal, suicidality, and psychosis reports: A disproportionality analysis of the Australian adverse events database

**Journal name**: *British Journal of Clinical Pharmacology*

**Author names**: Amy G. McNeilage, Ali Gholamrezaei, Bridin Murnion, Suzanne Nielsen, Claire E. Ashton-James

**Corresponding author**: Amy G. McNeilage (Sydney Medical School, Faculty of Medicine and Health, The University of Sydney). Address: Pain Management and Research Centre, Ground Floor, Douglas Building, Royal North Shore Hospital, St Leonards, NSW Australia 2065. Tel.: +61294631528. Email: amy.mcneilage@sydney.edu.au

**Resource 1:** Completed READUS-PV checklist

**Resource 2:** All pregabalin, gabapentin, duloxetine, and amitriptyline trade names included in extracted adverse event reports

**Resource 3:** Narrow and broad preferred terms included for each Standardised MedDRA Queries

**Resource 4:** Results of disproportionality analyses with both ROR and PRR

**Resource 5:** Explanation of the variables used in the calculation of ROR and PRR

**Resource 6:** Results of all sensitivity analyses (provided separately in .zip folder)

**Resource 1: Completed READUS-PV checklist**

| **Section and topic** | **Item #** | **Checklist item** | **Location where item is reported** |
| --- | --- | --- | --- |
| **Title** |  |  |  |
|  | ***1a*** | ***If disproportionality analyses are a prominent component of the published study, the study should be identified as a “disproportionality analysis”. The type of data and name of the database(s) should be specified.*** | P1 |
|  | ***1b*** | ***Report the name of adverse event(s) and/or drug(s) under study, when applicable.*** | P1 |
| **Introduction** |  |  |  |
| **Background** | ***2a*** | ***Describe the drug(s) and its utilization, the nature of the adverse event(s) under study and its frequency, and the existing knowledge on the drug-event combination.*** | P5,7-8 |
|  | ***2b*** | ***Specify the rationale for performing the analysis, e.g., as part of routine pharmacovigilance, to investigate an overall safety profile, or to assess a pre-specified hypothesis.*** | P6 |
|  | ***2c*** | ***Explain why ICSR databases and disproportionality analysis are suitable to fill the knowledge gap.*** | P6 |
| **Objectives** | ***3*** | ***State specific objectives, identifying the adverse event(s), the drug(s), and the reference group, including any pre-specified hypothesis, if applicable.*** | P6 |
| **Methods** |  |  |  |
| **Study design** | ***4a*** | ***Identify the study (i.e., “disproportionality analysis”) and the type of data used (e.g., “individual case safety reports”).*** | P6 |
|  | ***4b*** | ***Provide an outline of the entire study design, including primary and sensitivity analyses performed, and other designs such as case-by-case analysis or literature review.*** | P9-10 |
| **Data description, access, and pre-processing** | ***5a*** | ***Specify the name of the database(s), the database(s) custodian, and the coverage. Specify the type/number of drugs included within the database and the thesaurus, taxonomies, or ontologies used for coding drugs and events.*** | P7 |
|  | ***5b*** | ***Specify the extraction dates and describe and justify all choices used for data pre-processing, including any data transformation or exclusion, if appropriate.*** | P8-9 |
| **Variables definition** | ***6a*** | ***Describe the study population, including any restriction.*** | P8 |
|  | ***6b*** | ***Describe the nature and the meaning of key variables assessed in the work.*** | P8-9 |
|  | ***6c*** | ***Specify and justify any grouping of drugs or events. For drugs, specify and justify whether active ingredients/trade names/salts were considered and/or the selected role.*** | P7-8 |
|  | ***6d*** | ***Describe any additional data source used, the type of data, and how they interact with ICSRs.*** | NA |
| **Statistical methods** | ***7a*** | ***Present any descriptive analysis performed, specifying variables investigated, statistical tests, and significance thresholds.*** | P9 |
|  | ***7b*** | ***Describe the measure(s) selected for the disproportionality analysis including any threshold used to identify signals of disproportionate reporting. Explain the reason for this choice if applicable.*** | P9 |
|  | ***7c*** | ***Clearly describe any sensitivity analysis and any tool to control confounding, including any restriction, subgroup, stratification, adjustment, or interaction.*** | P10 |
|  | ***7d*** | ***Specify the variables and methods used for the case-by-case analysis, including any algorithm or criteria used to assess causality, if performed.*** | NA |
|  | ***7e*** | ***Specify any statistical methods used for other data sources.*** | NA |
| **Results** |  |  |  |
| **Participants** | ***8a*** | ***Specify the number of individual case safety reports included at each stage, including reasons for exclusion.*** | P10 |
|  | ***8b*** | ***Provide key demographic and clinical characteristics of cases, if possible comparing cases with any appropriate reference group.*** | P10 |
| **Disproportionality analysis** | ***9*** | ***Present all results including confidence intervals. Present also results of sensitivity analyses, if performed.*** | P11-13, SI |
| **Case-by-case analysis** | ***10*** | ***Present the case-by-case analysis of key variables. Present the causality assessment, if applicable.*** | NA |
| **Discussion** |  |  |  |
| **Key results** | ***11*** | ***Discuss key results with reference to study objectives and contextualize them within the current literature and other consulted sources. Clearly discriminate between expected reactions and emerging safety signals.*** | P13-14 |
| **External validity** | ***12a*** | ***Discuss the external validity of the results to the general population.*** | P14-16 |
|  | ***12b*** | ***Discuss the potential relevance of results in clinical practice*** | P14-16 |
|  | ***12c*** | ***Propose further study designs if applicable*** | P14, 16 |
| **Limitations** | ***13*** | ***Present general limitations, making clear that disproportionality analysis alone cannot prove causation or measure incidence, and specific limitations, including confounding and reporting bias and efforts to mitigate them.*** | P15 |
| **Declarations** |  |  |  |
|  | ***14a*** | ***Provide the source of funding/sponsorship and the role of the funders/sponsors for the present study and for any original study on which the present article is based.*** | P4 |
|  | ***14b*** | ***Clearly identify potential commercial and intellectual conflicts of interest (e.g., link to any drug/event investigated, whether financial, legal action, or software used).*** | P4 |
|  | ***14c*** | ***Declare any institutional approval needed or granted in the investigation.*** | P4 |
|  | ***14d*** | ***Include a statement on data availability, code availability (including the version of the statistical software used), and protocol registration.*** | P4 |

**Resource 2: All pregabalin, gabapentin, duloxetine, and amitriptyline trade names included in extracted adverse event reports**

| Active ingredient | Trade name |
| --- | --- |
| Pregabalin | APO-Pregabalin |
|  | Lypralin |
|  | Lyrica |
|  | Lyzalon |
|  | NEUROCCORD |
|  | PREGABALIN APOTEX |
|  | Pregabalin Sandoz |
| Gabapentin | APO-Gabapentin |
|  | Apotex Gabapentin |
|  | APX-GABAPENTIN |
|  | DBL Gabapentin |
|  | Gabacor |
|  | Gabahexal |
|  | Gabapentin Sandoz |
|  | Gabaran |
|  | Gabatine 300 |
|  | Gabatine 400 |
|  | Gantin |
|  | Gabapentin 300mg |
|  | GenRx Gabapentin |
|  | Neurontin |
|  | Nupentin |
|  | Nupentin 100 |
|  | Nupentin 300 |
|  | Nupentin 400 |
|  | Pharmacor Gabapentin |
| Duloxetine | Andepra |
|  | APO-Duloxetine |
|  | Apotex-Duloxetine |
|  | Coperin |
|  | Cymbalta |
|  | Deotine |
|  | DULOXETINE SANDOZ |
|  | Dytrex 30 |
|  | Dytrex 60 |
|  | Tixol |
| Amitriptyline | Amitriptyline (Mylan) |
|  | Amitriptyline Alphapharm |
|  | Amitriptyline Viatris |
|  | APO-Amitriptyline |
|  | APX-Amitriptyline |
|  | Endep |
|  | Endep 10 |
|  | Entrip |
|  | Tryptanol |

**Resource 3: Narrow and broad preferred terms included for each Standardised MedDRA Queries**

**Drug abuse and dependence (sub-SMQ) narrow terms**

1. Caffeine dependence
2. Dopamine dysregulation syndrome
3. Drug abuse
4. Drug abuser
5. Drug dependence
6. Drug dependence, antepartum
7. Drug dependence, postpartum
8. Drug use disorder
9. Drug use disorder, antepartum
10. Drug use disorder, postpartum
11. Intentional device misuse
12. Intentional overdose
13. Intentional product misuse
14. Maternal use of illicit drugs
15. Neonatal complications of substance abuse
16. Substance abuse
17. Substance abuser
18. Substance dependence
19. Substance use disorder

**Drug abuse and dependence (sub-SMQ) broad terms**

1. Caffeine dependence
2. Dopamine dysregulation syndrome
3. Drug abuse
4. Drug abuser
5. Drug dependence
6. Drug dependence, antepartum
7. Drug dependence, postpartum
8. Drug use disorder
9. Drug use disorder, antepartum
10. Drug use disorder, postpartum
11. Intentional device misuse
12. Intentional overdose
13. Intentional product misuse
14. Maternal use of illicit drugs
15. Neonatal complications of substance abuse
16. Substance abuse
17. Substance abuser
18. Substance dependence
19. Substance use disorder
20. Accidental overdose
21. Cannabinoid hyperemesis syndrome
22. Delusion of parasitosis
23. Dependence
24. Disturbance in social behaviour
25. Drug detoxification
26. Drug diversion
27. Drug level above therapeutic
28. Drug level increased
29. Drug screen
30. Drug screen positive
31. Drug tolerance
32. Drug tolerance decreased
33. Drug tolerance increased
34. Illicit prescription attainment
35. Incorrect route of product administration
36. Intentional product use issue
37. Medication overuse headache
38. Multiple use of single-use product
39. Narcotic bowel syndrome
40. Needle track marks
41. Overdose
42. Performance enhancing product use
43. Pharmaceutical nomadism
44. Prescribed overdose
45. Prescription drug used without a prescription
46. Prescription form tampering
47. Product administered at inappropriate site
48. Reversal of opiate activity
49. Substance use
50. Substance-induced mood disorder
51. Substance-induced psychotic disorder
52. Toxicity to various agents

**Drug withdrawal (sub-SMQ) narrow terms**

1. Drug withdrawal convulsions
2. Drug withdrawal headache
3. Drug withdrawal maintenance therapy
4. Drug withdrawal syndrome
5. Drug withdrawal syndrome neonatal

**Drug withdrawal (sub-SMQ) broad terms**

1. Drug withdrawal convulsions
2. Drug withdrawal headache
3. Drug withdrawal maintenance therapy
4. Drug withdrawal syndrome
5. Drug withdrawal syndrome neonatal
6. Antidepressant discontinuation syndrome
7. Cholinergic rebound syndrome
8. Delusion of parasitosis
9. Dopamine agonist withdrawal syndrome
10. Drug rehabilitation
11. Rebound effect
12. Steroid withdrawal syndrome
13. Topical steroid withdrawal reaction
14. Withdrawal arrhythmia
15. Withdrawal catatonia
16. Withdrawal syndrome

**Suicide and self-injury (sub-SMQ) narrow terms**

*This SMQ includes narrow terms only*

1. Assisted suicide
2. Columbia suicide severity rating scale abnormal
3. Completed suicide
4. Depression suicidal
5. Intentional overdose
6. Intentional self-injury
7. Poisoning deliberate
8. Self-injurious ideation
9. Suicidal behaviour
10. Suicidal ideation
11. Suicide attempt
12. Suicide threat
13. Suspected suicide
14. Suspected suicide attempt

**Psychosis and psychotic disorders (SMQ) narrow terms**

1. Acute psychosis
2. Alcoholic psychosis
3. Alice in wonderland syndrome
4. Brief psychotic disorder with marked stressors
5. Brief psychotic disorder without marked stressors
6. Brief psychotic disorder, with postpartum onset
7. Charles Bonnet syndrome
8. Childhood psychosis
9. Clang associations
10. Cotard's syndrome
11. Delusion
12. Delusion of grandeur
13. Delusion of parasitosis
14. Delusion of reference
15. Delusion of replacement
16. Delusion of theft
17. Delusional disorder, erotomanic type
18. Delusional disorder, grandiose type
19. Delusional disorder, jealous type
20. Delusional disorder, mixed type
21. Delusional disorder, persecutory type
22. Delusional disorder, somatic type
23. Delusional disorder, unspecified type
24. Delusional perception
25. Dementia of the Alzheimer's type, with delusions
26. Depressive delusion
27. Derailment
28. Dopamine supersensitivity psychosis
29. Epileptic psychosis
30. Erotomanic delusion
31. Flight of ideas
32. Hallucination
33. Hallucination, auditory
34. Hallucination, gustatory
35. Hallucination, olfactory
36. Hallucination, synaesthetic
37. Hallucination, tactile
38. Hallucination, visual
39. Hallucinations, mixed
40. Hypnagogic hallucination
41. Hypnopompic hallucination
42. Hysterical psychosis
43. Ideas of reference
44. Illusion
45. Jealous delusion
46. Loose associations
47. Mixed delusion
48. Negative symptoms in schizophrenia
49. Neologism
50. Neuroleptic-induced deficit syndrome
51. Paranoia
52. Paranoid personality disorder
53. Parkinson's disease psychosis
54. Paroxysmal perceptual alteration
55. Persecutory delusion
56. Post-injection delirium sedation syndrome
57. Postictal psychosis
58. Posturing
59. Pseudohallucination
60. Psychosis postoperative
61. Psychotic behaviour
62. Psychotic disorder
63. Psychotic disorder due to a general medical condition
64. Reactive psychosis
65. Rebound psychosis
66. Schizoaffective disorder
67. Schizoaffective disorder bipolar type
68. Schizoaffective disorder depressive type
69. Schizophrenia
70. Schizophreniform disorder
71. Schizotypal personality disorder
72. Senile psychosis
73. Shared psychotic disorder
74. Somatic delusion
75. Somatic hallucination
76. Substance-induced psychotic disorder
77. Tangentiality
78. Thought blocking
79. Thought broadcasting
80. Thought insertion
81. Thought withdrawal
82. Transient psychosis
83. Waxy flexibility

**Psychosis and psychotic disorders (SMQ) broad terms**

1. Acute psychosis
2. Alcoholic psychosis
3. Alice in wonderland syndrome
4. Brief psychotic disorder with marked stressors
5. Brief psychotic disorder without marked stressors
6. Brief psychotic disorder, with postpartum onset
7. Charles Bonnet syndrome
8. Childhood psychosis
9. Clang associations
10. Cotard's syndrome
11. Delusion
12. Delusion of grandeur
13. Delusion of parasitosis
14. Delusion of reference
15. Delusion of replacement
16. Delusion of theft
17. Delusional disorder, erotomanic type
18. Delusional disorder, grandiose type
19. Delusional disorder, jealous type
20. Delusional disorder, mixed type
21. Delusional disorder, persecutory type
22. Delusional disorder, somatic type
23. Delusional disorder, unspecified type
24. Delusional perception
25. Dementia of the Alzheimer's type, with delusions
26. Depressive delusion
27. Derailment
28. Dopamine supersensitivity psychosis
29. Epileptic psychosis
30. Erotomanic delusion
31. Flight of ideas
32. Hallucination
33. Hallucination, auditory
34. Hallucination, gustatory
35. Hallucination, olfactory
36. Hallucination, synaesthetic
37. Hallucination, tactile
38. Hallucination, visual
39. Hallucinations, mixed
40. Hypnagogic hallucination
41. Hypnopompic hallucination
42. Hysterical psychosis
43. Ideas of reference
44. Illusion
45. Jealous delusion
46. Loose associations
47. Mixed delusion
48. Negative symptoms in schizophrenia
49. Neologism
50. Neuroleptic-induced deficit syndrome
51. Paranoia
52. Paranoid personality disorder
53. Parkinson's disease psychosis
54. Paroxysmal perceptual alteration
55. Persecutory delusion
56. Post-injection delirium sedation syndrome
57. Postictal psychosis
58. Posturing
59. Pseudohallucination
60. Psychosis postoperative
61. Psychotic behaviour
62. Psychotic disorder
63. Psychotic disorder due to a general medical condition
64. Reactive psychosis
65. Rebound psychosis
66. Schizoaffective disorder
67. Schizoaffective disorder bipolar type
68. Schizoaffective disorder depressive type
69. Schizophrenia
70. Schizophreniform disorder
71. Schizotypal personality disorder
72. Senile psychosis
73. Shared psychotic disorder
74. Somatic delusion
75. Somatic hallucination
76. Substance-induced psychotic disorder
77. Tangentiality
78. Thought blocking
79. Thought broadcasting
80. Thought insertion
81. Thought withdrawal
82. Transient psychosis
83. Waxy flexibility
84. Abnormal behaviour
85. Abulia
86. Affect lability
87. Affective ambivalence
88. Affective disorder
89. Alcohol withdrawal syndrome
90. Anosognosia
91. Apathy
92. Asocial behaviour
93. Behaviour disorder
94. Bipolar I disorder
95. Blunted affect
96. Bradyphrenia
97. Catatonia
98. Constricted affect
99. Disorganised speech
100. Dyslogia
101. Echolalia
102. Echopraxia
103. Emotional poverty
104. Flat affect
105. Grandiosity
106. Hypomania
107. Idioglossia
108. Illogical thinking
109. Impaired reasoning
110. Inappropriate affect
111. Incoherent
112. Intrusive thoughts
113. Lack of spontaneous speech
114. Logorrhoea
115. Magical thinking
116. Major depression
117. Malignant catatonia
118. Mania
119. Manic symptom
120. Mutism
121. Obsessive rumination
122. Paralogism
123. Perseveration
124. Poverty of speech
125. Poverty of thought content
126. Presenile dementia
127. Pressure of speech
128. Senile dementia
129. Social avoidant behaviour
130. Speech disorder
131. Suspiciousness
132. Tachyphrenia
133. Thinking abnormal
134. Vascular dementia
135. Verbigeration
136. Wernicke-Korsakoff syndrome
137. Withdrawal catatonia

**Resource 4: Results of disproportionality analyses with both ROR and PRR**

Results of active comparator disproportionality analyses for AEs of interest

| AE of interest | Pregabalin | | Other neuropathic pain drugs | |
| --- | --- | --- | --- | --- |
|  | ROR (95% CI) | PRR (95% CI) | ROR (95% CI) | PRR (95% CI) |
| Drug abuse and dependence | 1.38 (1.17, 1.62)* | 1.30 (1.14, 1.49)* | REF | REF |
| Drug withdrawal | 0.46 (0.32, 0.67)* | 0.47 (0.33, 0.68)* | REF | REF |
| Suicide and self-injury | 1.09 (0.88, 1.34) | 1.08 (0.89, 1.30) | REF | REF |
| Psychosis and psychotic disorders | 1.26 (1.002, 1.57)* | 1.23 (1.002, 1.52)* | REF | REF |

*Note*: * indicates *p* < .05. REF = reference set. Other neuropathic pain drugs set includes gabapentin, duloxetine, and amitriptyline combined.

Results of sensitivity analyses using different comparators

| AE of interest | Comparator drug | Pregabalin | |
| --- | --- | --- | --- |
|  |  | ROR (95% CI) | PRR (95% CI) |
| Drug abuse and dependence | Gabapentin | 2.92 (2.03, 4.20)* | 2.52 (1.82, 3.51)* |
|  | Duloxetine | 1.83 (1.48, 2.27)* | 1.66 (1.38, 1.99)* |
|  | Amitriptyline | 0.64 (0.52, 0.78)* | 0.71 (0.61, 0.83)* |
| Drug withdrawal | Gabapentin | 1.34 (0.60, 3.00) | 1.33 (0.60, 2.93) |
|  | Duloxetine | 0.27 (0.19, 0.40)* | 0.29 (0.20, 0.42)* |
|  | Amitriptyline | 1.80 (0.81, 4.03) | 1.79 (0.81, 3.95) |
| Suicide and self-injury | Gabapentin | 1.77 (1.16, 2.70)* | 1.69 (1.14, 2.51)* |
|  | Duloxetine | 0.82 (0.65, 1.04) | 0.84 (0.68, 1.03) |
|  | Amitriptyline | 1.35 (0.96, 1.89) | 1.31 (0.97, 1.79) |
| Psychosis and psychotic disorders | Gabapentin | 1.04 (0.72, 1.51) | 1.04 (0.75, 1.45) |
|  | Duloxetine | 1.59 (1.78, 2.14)* | 1.53 (1.16, 2.03)* |
|  | Amitriptyline | 1.00 (0.73, 1.38) | 1.00 (0.75, 1.34) |

*Note*: * indicates *p* < .05.

**Resource 5: Explanation of the variables used in the calculation of ROR and PRR**

|  | AE of interest | Other AEs | All AEs |
| --- | --- | --- | --- |
| Pregabalin | A | B | A+B |
| Control drugs | C | D | C+D |

*Note*: each cell represents the number of associated case reports in the database
